# Supplementary material for: Urban population structure and dispersal of an Australian mosquito (Aedes notoscriptus) involved in disease transmission
Source: Heredity (Edinb). 2022 Dec 20;130(2):99–108. doi: 10.1038/s41437-022-00584-4 (PMC9905534; doi:10.1038/s41437-022-00584-4)
Supplement: Supplementary file 1 — Supplementary Information [file 41437_2022_584_MOESM1_ESM.pdf]

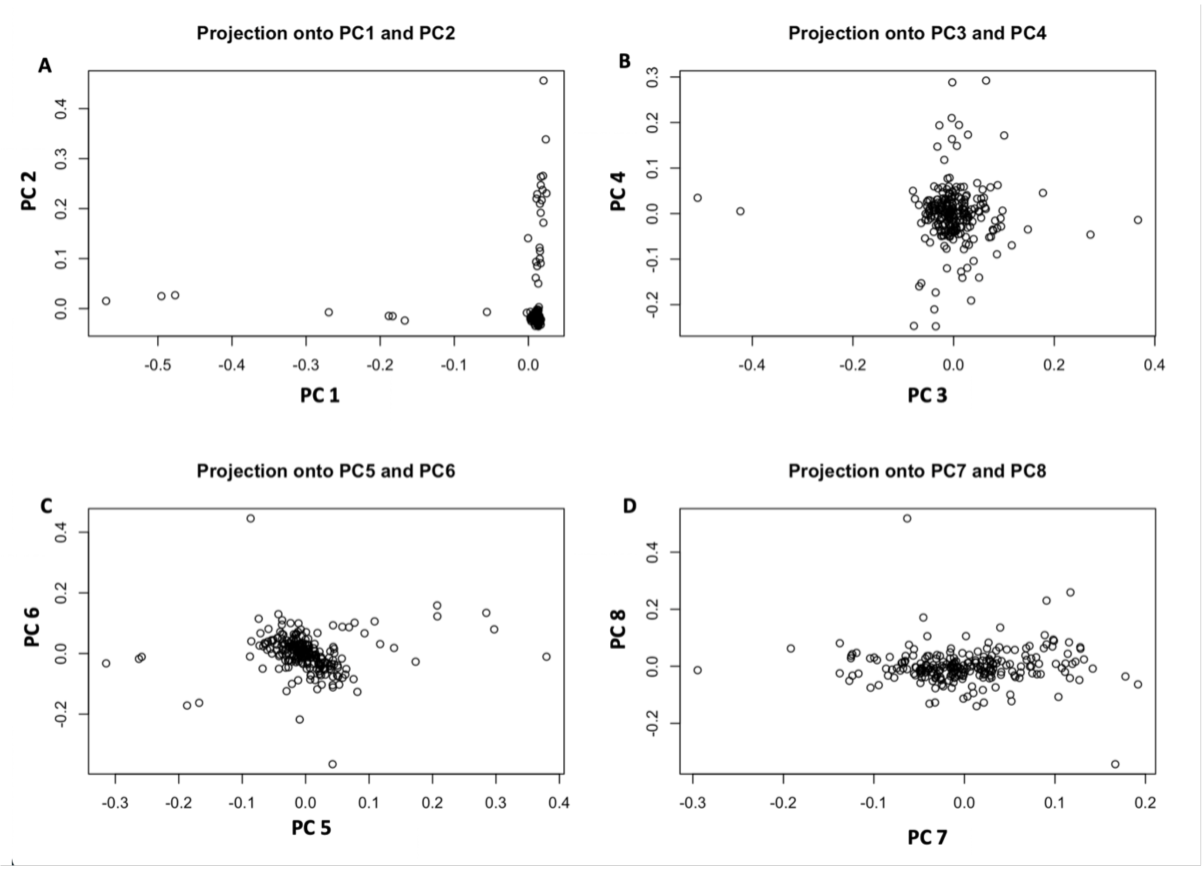


**Figure S1** ***Genome-wide genetic structure of all samples for different PC conditions.*** *(A) Projection onto the first and second PCs. (B) Third and fourth PCs. (C) Fifth and sixth PCs. (D) Seventh and eighth PCs.*

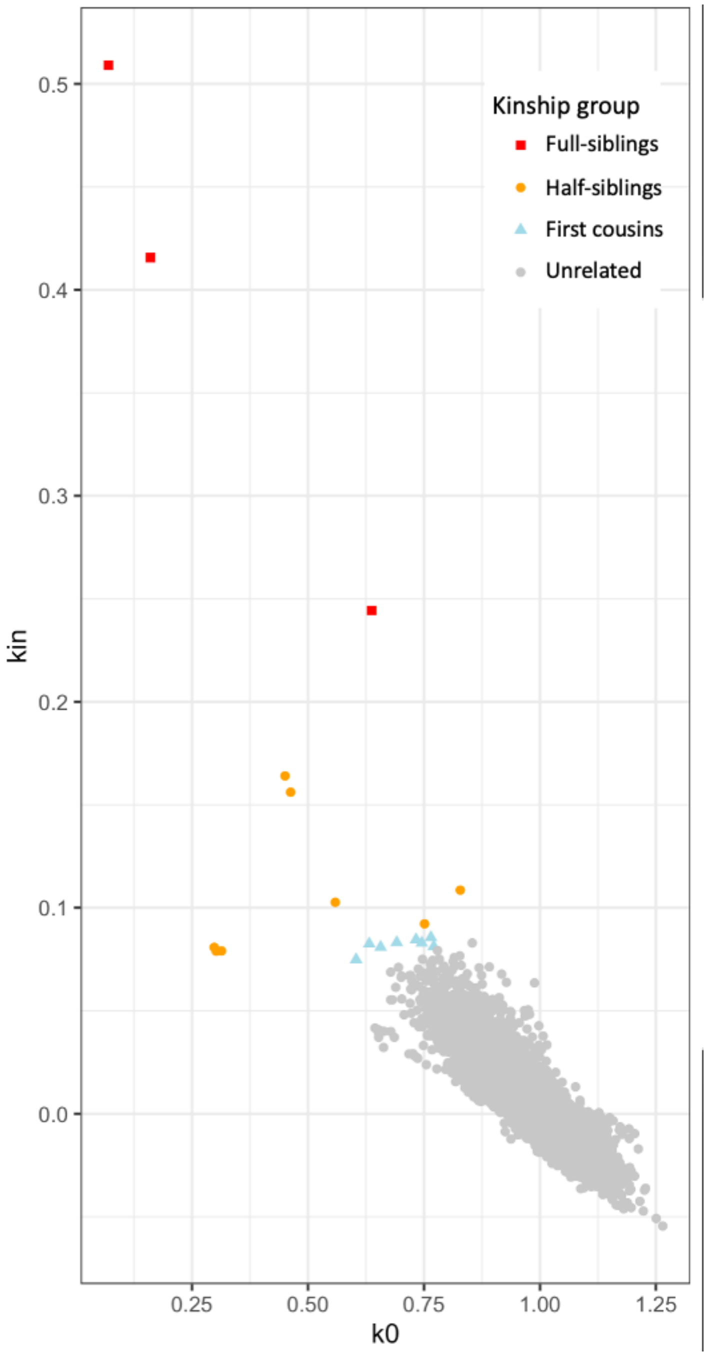


**Figure S2** ***Scatterplot of the estimated kinship coefficients (Kin) and estimated probabilities of sharing zero alleles IBD*** *(k0)*. Red squares represent full-siblings, yellow circles half-siblings, blue triangles first cousins and grey circles unrelated individuals.

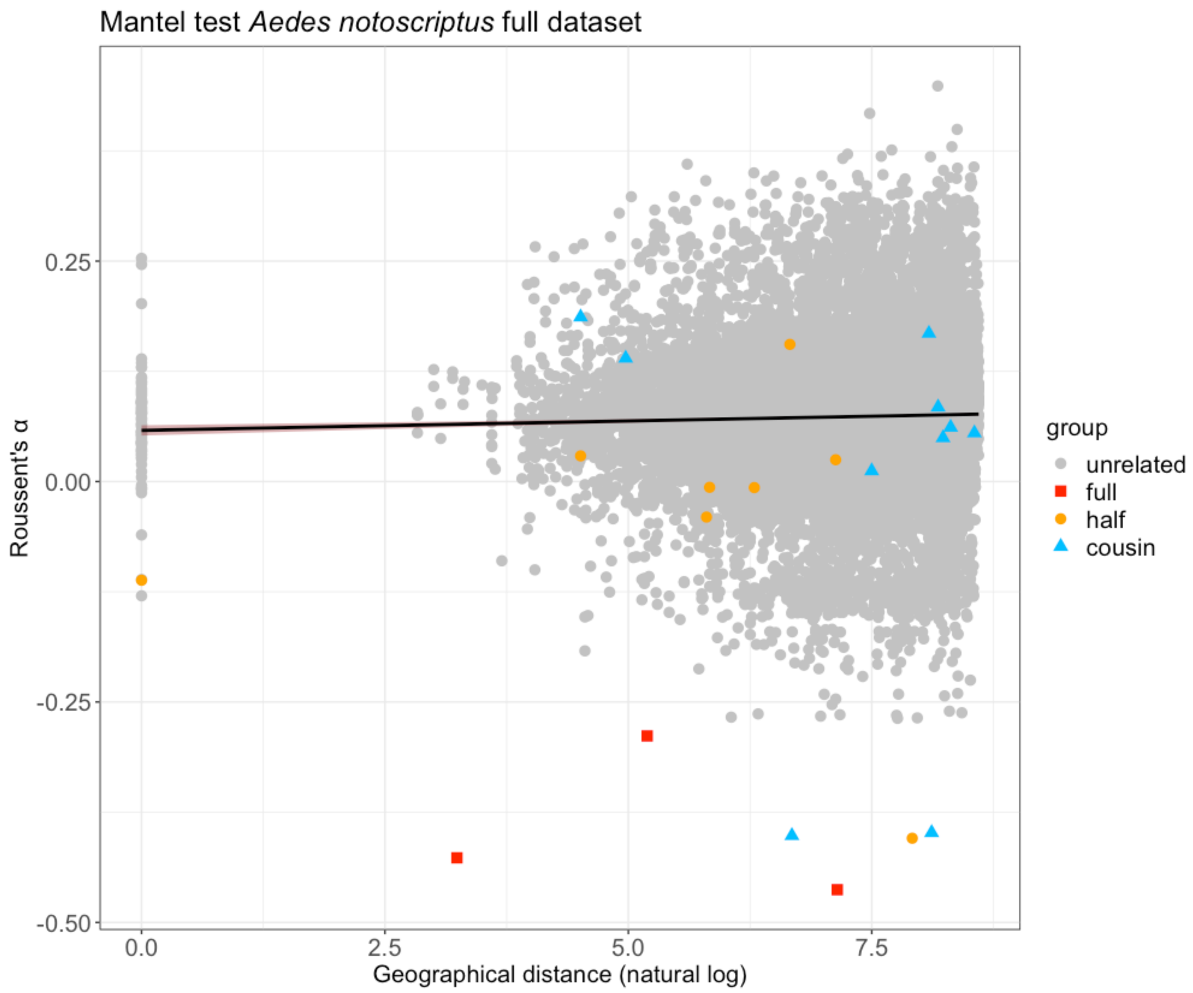


**Figure S3: Scatter plot of a mantel test between geographic (natural log) and genetic distance (Rousset's a) for the full *Aedes notoscriptus* dataset**. Full siblings are shown as red squares, half-siblings as orange dots, first cousins as blue triangles and unrelated individuals as grey dots. Line describes a linear regression fit. n = 240 individuals.

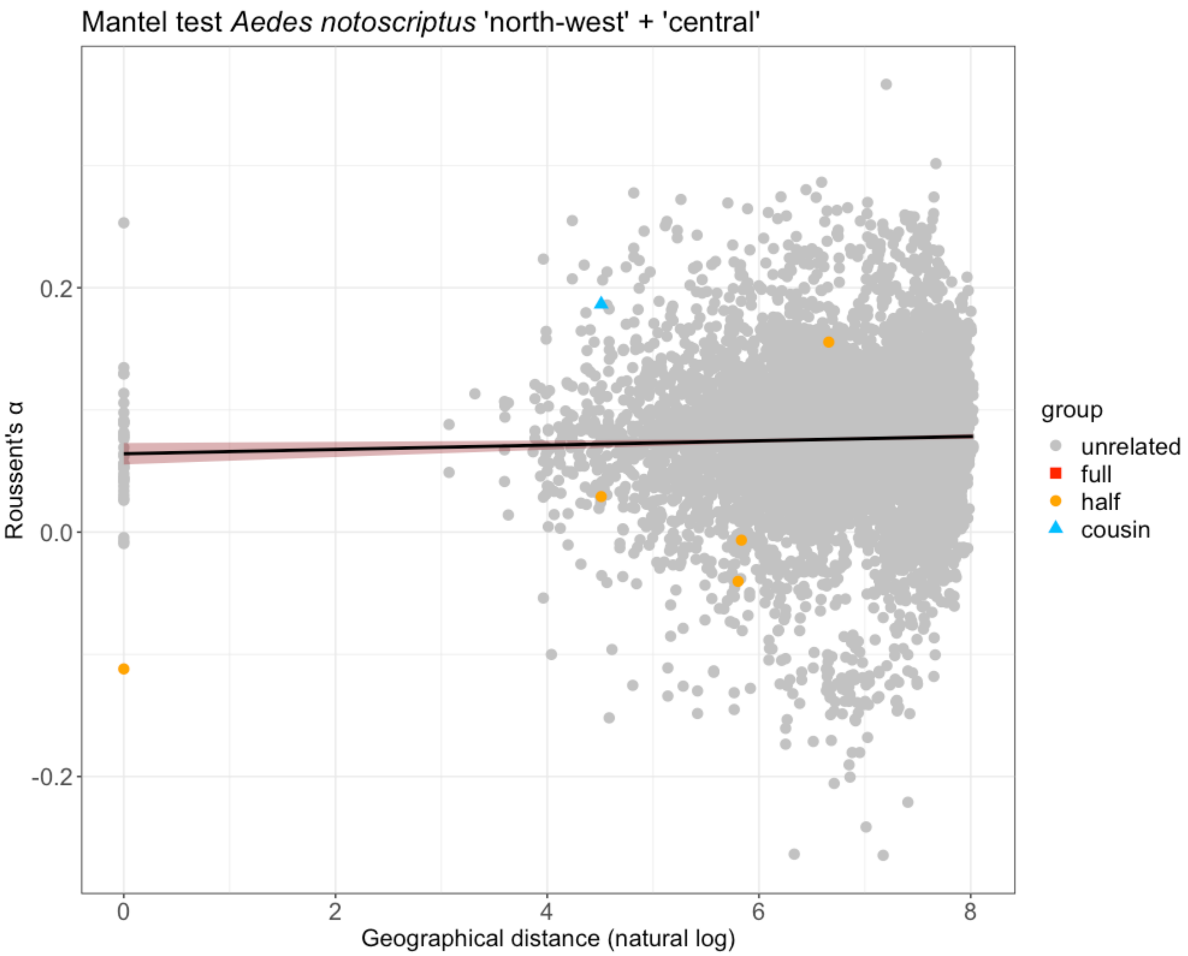


***Figure S4: Scatter plot of a mantel test between geographic (natural log) and genetic distance (Rousset's a) for the 'north-west' + 'central' Aedes notoscriptus dataset.*** *Full siblings are shown as red squares, half-siblings as orange dots, first cousins as blue triangles and unrelated individuals as grey dots. Line describes a linear regression fit. n = 128 individuals.*

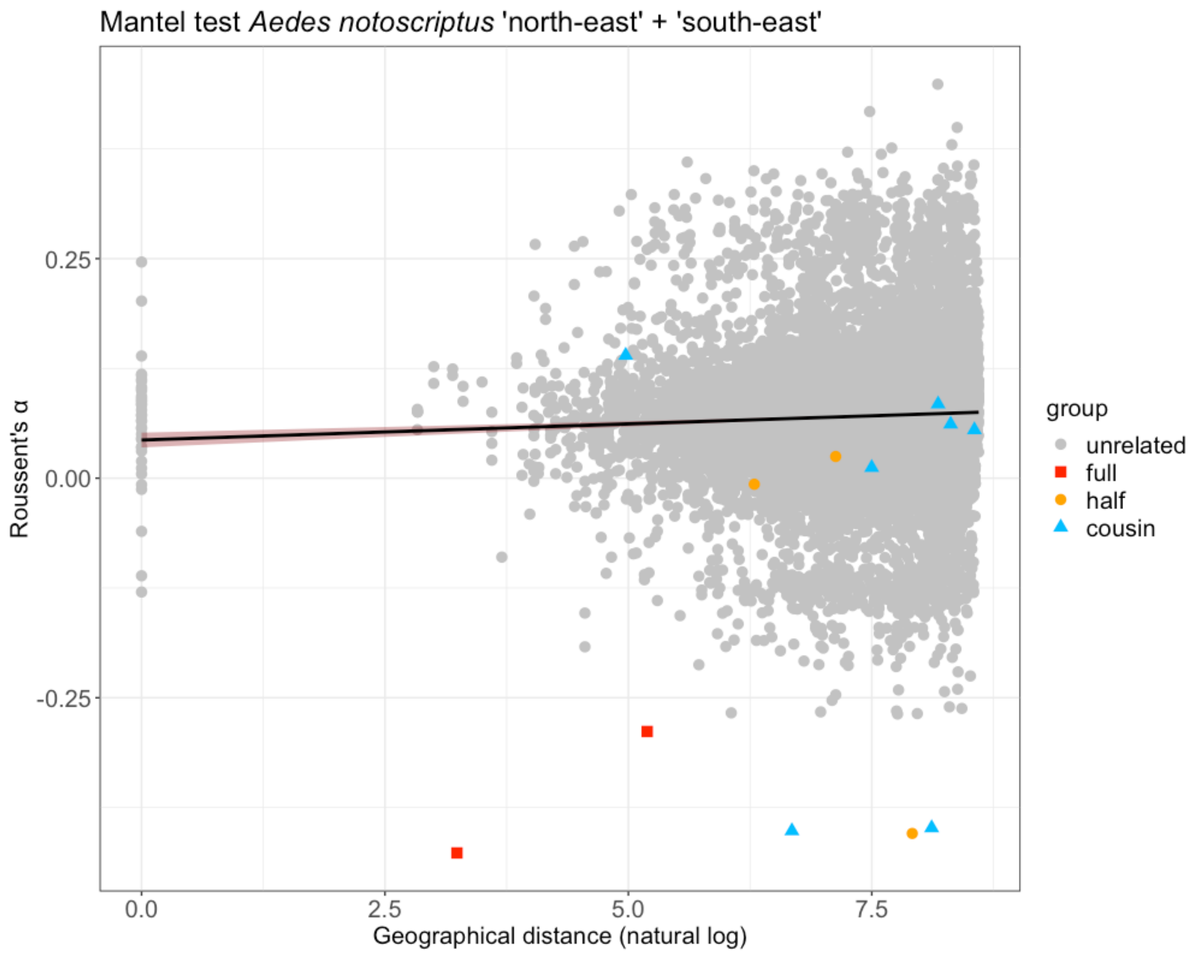


***Figure S5: Scatter plot of a mantel test between geographic (natural log) and genetic distance (Rousset's a) for the 'north-east + 'south-east' Aedes notoscriptus dataset.*** *Full siblings are shown as red squares, half-siblings as orange dots, first cousins as blue triangles and unrelated individuals as grey dots. Line describes a linear regression fit. n = 112 individuals.*

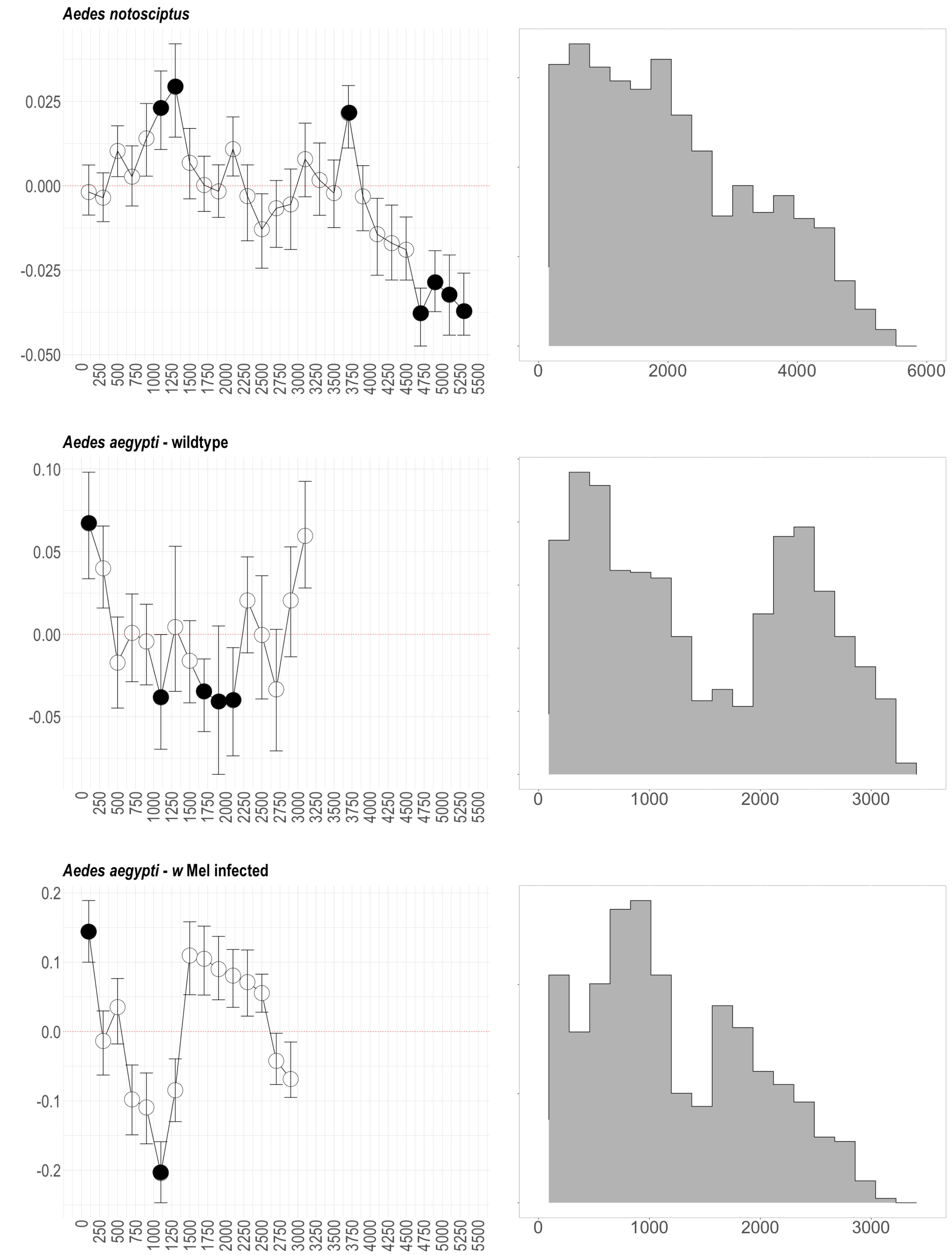


***Figure S6 Spatial autocorrelation (left) and density of trap distances (right)*** *of* Aedes notoscriptus ***(A)****, WT (wild type = uninfected)* Aedes aegypti ***(B)*** *and* w*Mel infected* Aedes aegypti ***(C)****. Mantel correlograms on the left depict the association between*

*genetic distance and geographical distance among pairs of the same distance class of 100m. Error bars show 95% confidence intervals and were calculated using 999 bootstrap replicates. Significant associations (α = 0.05) are shown as filled circles. Density histograms on the right describe the pairwise distances between traps of the three datasets.* Ae. aegypti *data is from Schmidt* et al. *(2018).*
